# Cosmogenic exposure dating reveals limited long-term variability in erosion of a rocky coastline

**DOI:** 10.1038/s41467-020-17611-9

**Published:** 2020-07-30

**Authors:** Zuzanna M. Swirad, Nick J. Rosser, Matthew J. Brain, Dylan H. Rood, Martin D. Hurst, Klaus M. Wilcken, John Barlow

**Affiliations:** 1 Department of Geography, Durham University, South Road, Durham, DH1 3LE UK; 2 Scripps Institution of Oceanography, University of California San Diego, 9500 Gilman Drive, La Jolla, CA 92093 USA; 3 Department of Earth Science and Engineering, Imperial College London, South Kensington Campus, London, SW7 2AZ UK; 4 School of Geographical and Earth Sciences, University of Glasgow, University Avenue, Glasgow, G12 8QQ UK; 5 Australian Nuclear Science and Technology Organization, New Illawarra Road, Lucas Heights, NSW 2234 Australia; 6 Department of Geography, University of Sussex, Brighton, BN1 9QJ UK

**Keywords:** Ocean sciences, Physical oceanography, Geochemistry, Geomorphology

## Abstract

Predicted sea-level rise and increased storminess are anticipated to lead to increases in coastal erosion. However, assessing if and how rocky coasts will respond to changes in marine conditions is difficult due to current limitations of monitoring and modelling. Here, we measured cosmogenic ^10^Be concentrations across a sandstone shore platform in North Yorkshire, UK, to model the changes in coastal erosion within the last 7 kyr and for the first time quantify the relative long-term eros0ive contribution of landward cliff retreat, and down-wearing and stripping of rock from the shore platform. The results suggest that the cliff has been retreating at a steady rate of 4.5 ± 0.63 cm yr^−1^, whilst maintaining a similar profile form. Our results imply a lack of a direct relationship between relative sea level over centennial to millennial timescales and the erosion response of the coast, highlighting a need to more fully characterise the spatial variability in, and controls on, rocky coast erosion under changing conditions.

## Abstract (web summary)

Predicted sea-level rise is widely anticipated to lead to increased coastal erosion, however, assessing how rocky coasts will respond to changes in marine conditions is difficult to constrain. Here, the authors find that a North Yorkshire rocky cliff has been eroding at a similar rate over the last 7 kyr, and they do not observe an increase in erosion rates in response to modern sea level rise.

## Introduction

Understanding the rate and nature of coastal erosion is pivotal in predicting future change under anticipated increases in sea level and storminess^1^. Existing models of rocky coast evolution are either conceptual^2,3^ or based on highly abstracted physics with coefficients that remain difficult to quantify^4–7^. It is problematic to make use of empirical data to constrain long-term erosion rates due to the low accuracy of cartographic maps relative to the often-low magnitudes of erosion, and the presently limited duration of monitoring of sufficient precision to detect change^8^. These factors may cause either under- or overestimation of long-term (centennial to millennial) erosion due to its putative episodic nature^9–11^. Moreover, lagged and indirect responses of rocky coasts to environmental conditions make it difficult to construct accurate process-based erosion models that can be validated^12^.

At present, the only way to verify the accuracy of models of long-term change of fully erosional rocky coasts is via cosmogenic radionuclide exposure dating^8,13^. Calculation of isotope concentrations across an active shore platform allows previous position(s) of the cliff to be reconstructed from their timing of exposure of the foreshore. Choi et al.^14^ were able to use this approach to confirm that the current coastal configuration in W Korea was inherited from a previous sea-level high stand, rather than being solely a consequence of current erosion. Regard et al.^15^ and Hurst et al.^16^ derived millennial rates of chalk-cliff retreat on two sides of the English Channel and compared these with contemporary rates calculated from maps to show either the similarity of the long-term and contemporary erosion rates^15^, or to suggest that rates of erosion have been more rapid during the last 150 years than for much of the Holocene^16^ at these two sites, respectively. Until now, studies that consider if this approach can be used to describe short-term variability in erosion over millennia, by identifying large-scale erosion events, as documented for the N English Channel^17^, or periods of heightened erosion rates that would allow any future change to be put into a long-term context, have not been undertaken.

The models that use cosmogenic isotope concentrations to derive long-term rocky coast erosion are based around fundamental assumptions of how relative sea-level (RSL) change and the shore platform interact to drive erosion of the cliff. These studies have by necessity simplified the erosion of shore platforms by assuming erosion rates to be negligible^14^ or pre-defining a geometry-based long-term erosion trend, such that platform lowering is proportional to cliff retreat rate where an equilibrium coastal profile is maintained (profile-parallel coastal erosion^3^)^15,16^. They have also simplified the potential role of mesoscale (10^−1^–10^0^ m) block detachment stripping material from the foreshore^18,19^. While the potentially highly important role of some of these factors in reconstructing the rate and nature of rocky coast erosion has been conceptually demonstrated^13^, this has yet to be explored with field data.

**Fig. 1:**
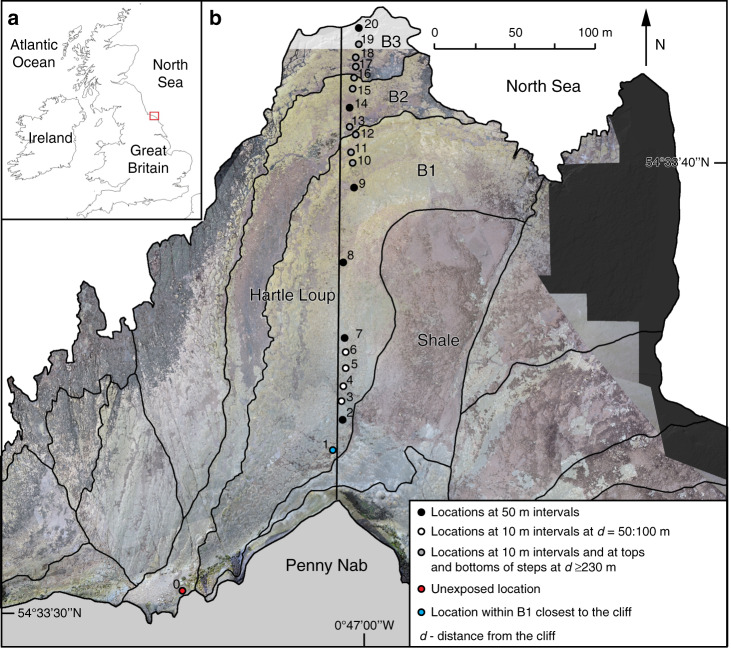
Maps of sampling sites. **a** Location of North Yorkshire on British Isles (red box). **b** Location of the 300-m profile (elevation distribution in Fig. 2a) and 21 sampling sites for exposure dating across the Hartle Loup shore platform; background: LiDAR-derived ortho-photomap, 2016 (for survey details, see Benjamin et al.^41^).

The aim of this study was to reconstruct the long-term history of rocky coast erosion rates using measurements of cosmogenic ^10^Be concentrations across an active shore platform. We studied a section of shore platform on the coast of the North York Moors National Park, UK (Fig. 1), a storm-dominated macro-tidal coast with a spring tide range of 4.6 m and a neap tide range of 2.25 m (http://www.ntslf.org/). The dominant wave direction is from the NE with the mean wave height of 0.95 m (max. 9.26 m), as reported for a location 1.5 km offshore of our study site near Staithes in 2013–2014^20^. RSL here has risen by ~5.8 m within the last 7 kyr^21,22^. Over this time period, RSL rise decelerated, with cessation of ice-sheet melt input to global sea level around 4 kyr BP^23,24^. Global rates of sea-level rise, and so potentially RSL at our site, increased at a rate of 0.6 mm yr^−1^ during the Medieval Climatic Anomaly (MCA, 800–1300 CE), and fell slightly (−0.1 mm yr^−1^) during the Little Ice Age (LIA, 1400–1850 CE)^25–27^. Between 1850 and 1900 CE, modern rates (2–3 mm yr^−1^) of global sea-level rise began^26^.

The 300-m-wide Hartle Loup shore platform has an average surface slope of 0.4°, and is formed in Jurassic shale and fine-grained sandstones, with a bedding dip 2° to the south–east^28^. The bedding dip results in three sandstone beds (B1–B3: Fig. 1b) exposed along the foreshore surface, which each terminate with abrupt breaks in slope, herein referred to as steps at the termini of beds B1 (0.80-m thick) and B2 (0.75-m thick), and a seaward edge at the terminus of B3^29^. Submerged layers of sedimentary rocks are present seawards from the platform edge, but are not considered here. The platform ends at the foot of an ~60-m-high cliff (Penny Nab) that retreats at the rate of 2.7 ± 2.9 cm yr^−1^ when monitored over 7 years^30^. To aid comparison to previous literature, cliff retreat and step back-wearing rates are henceforth provided in cm yr^−1^, while shore platform down-wearing is reported in mm yr^−1^.

Down-wearing rates on shore platforms along the North Yorkshire coast measured with a micro-erosion meter averaged 3.21 ± 4.76 mm yr^−1^ ranging from −1.5 mm yr^−1^ (surface swelling) to 19.31 mm yr^−1^ (where shallow beach deposits were available for abrasion) between 70 monitoring sites^31^. Swirad et al.^32^ obtained an order-of-magnitude lower average rate of 0.528 ± 0.088 mm yr^−1^ for the Hartle Loup platform by using wider coverage Structure-from-Motion. The down-wearing rates were found to be higher landwards and at locations with more tidal emergence/submergence cycles. Spatial variation also occurred across rock types; the average sandstone down-wearing rate was 0.222 ± 0.122 mm yr^−1^, which was slower than the down-wearing rate of 0.682 ± 0.336 mm yr^−1^ in shale^32^.

In this study, we first measured cosmogenic ^10^Be concentrations. We collected sandstone samples from 20 locations along a single-shore platform transect and from one location at the toe of the cliff face, henceforth referred to as the unexposed sample (#0; 0 m) (Fig. 1). We processed the samples in the laboratory and measured ^10^Be concentrations (Supplementary Note 1; Supplementary Data 1; Supplementary Tables 1 and 2). We then modelled ^10^Be concentrations using a range of cliff retreat and shore platform down-wearing scenarios. We selected the scenarios that best matched measured ^10^Be concentrations. Finally, we used the difference between measured and modelled concentrations to calculate step back-wearing rates.

The results of our numerical modelling suggest that the cliff has retreated at the steady rate of 4.5 ± 0.63 cm yr^−1^ over the last 7 kyr, which is similar to observed contemporary, short-term rates^30^, and implies a lack of a direct relationship between cliff retreat and RSL rise. Long-term shore platform down-wearing rates are controlled by rates of cliff retreat and RSL rise. Step back-wearing on the shore platform represents 14.6% of long-term foreshore erosion. Our data highlight the multifaceted character of rocky coast erosion that should be reflected in modelling to predict future change.

## Results

### Measured and modelled ^10^Be concentrations

**Fig. 2:**
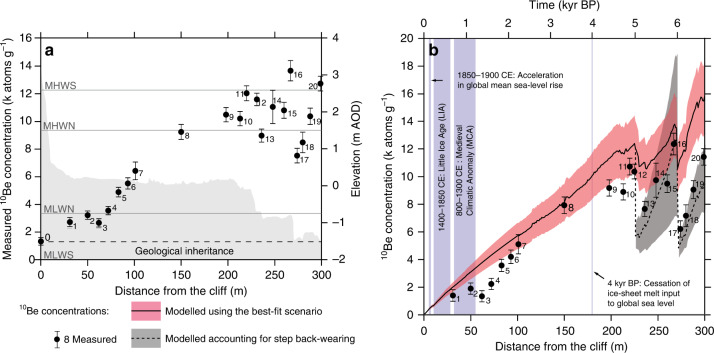
Across-shore distribution of cosmogenic ^10^Be concentrations. **a** Raw measured concentrations ± AMS measurement error and background uncertainty propagating in quadrature (Supplementary Note 1); dashed line delimits concentration due to deep muon production (geological inheritance); grey shadow (secondary axis) represents the elevation distribution across the profile indicated in Fig. 1b; grey lines indicate tidal levels: MHWS = 2.59-m AOD: mean high water level of spring tides; MHWN = 1.50-m AOD: mean high water level of neap tides; MLWN = −0.75-m AOD: mean low water level of neap tides; MLWS = −2.01-m AOD: mean low water level of spring tides (https://www.ntslf.org/). **b** Measured concentrations corrected for geological inheritance (concentration at #0 subtracted from raw measurements) ± AMS measurement error and background- and inheritance uncertainties propagating in quadrature (Supplementary Note 1) and those modelled using the scenario of steady cliff retreat rate of 4.5 ± 0.63 cm yr^−1^ and profile-parallel coastal erosion (solid line and red error envelope). Dashed line and grey error envelope represent modelled concentrations, including the rate of step back-wearing (mean ± standard deviation). Vertical blue lines and boxes indicate major influences on relative sea level at the study location.

^10^Be concentrations ranged between 1304 ± 268 atoms g^−1^ in the unexposed sample (#0) and 13,630 ± 781 atoms g^−1^ (#16; 267 m) (Fig. 2a). The concentrations increased gradually with distance seaward, with two abrupt drops in concentration at topographic steps at the transition between stratigraphic beds at the B1/B2 boundary (#12–13; 231–236 m), and at the B2/B3 boundary (#16–17; 267–274 m). The ^10^Be concentrations in some samples were relatively low compared with adjacent samples (#3; 62 m and #10; 213 m) (Fig. 2a).

We developed an inverse model to predict ^10^Be concentrations as a function of cliff retreat rates and platform erosion assuming (i) constant cliff shape, (ii) zero-surface reburial after initial exposure on the foreshore, (iii) zero shielding from ephemeral beach cover and (iv) a constant wave climate and tidal regime. We limited the period of interest to the last 7 kyr when RSL has been at elevations sufficient to drive erosion of the present-day coast. We quantified the impact of topography, water shielding and temporal variability in the incoming cosmic-ray flux using existing models^33–36^. We modelled 232 scenarios that considered how different combinations of rates and trends of cliff retreat (ranging from a steady rate through to a linear acceleration or deceleration) and shore platform down-wearing models (zero-surface down-wearing, profile-parallel coastal erosion^3^, platform widening^2^ and an empirically based model^32^) influenced predicted concentrations of ^10^Be along the foreshore transect.

We then compared the modelled outputs with our measured concentrations of ^10^Be to infer the most likely scenarios of cliff retreat at our study site. We selected the most likely scenarios of past coastal erosion according to their match with measured ^10^Be concentrations using three criteria (see “Methods”): (i) the modelled exposure of the whole platform profile within the last 7 kyr, (ii) statistical similarity: the normalised root-mean-square deviation (NRMSD) < 0.2 (subjective threshold of acceptability) between measured and modelled concentrations of non-stepped section of the profile (#1–12; ≤231 m) and (iii) modelled concentrations that were higher or equal to measured concentrations at locations seawards of steps (#13–20; ≥236 m). The third criterion is based on the assumption that if sites located on the lower rock beds (B2–B3) were affected by both surface down-wearing and block removal, the measured concentrations would be lower than those modelled using the down-wearing only.

**Fig. 3:**
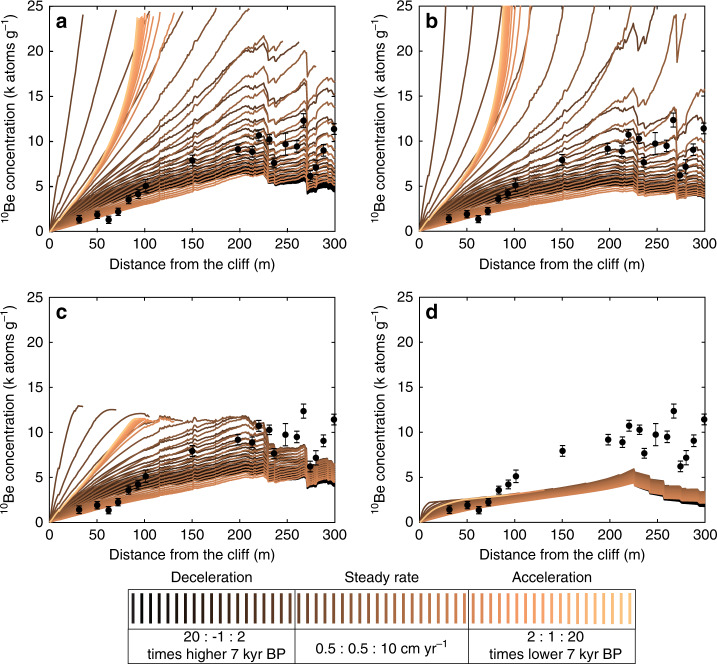
Results of modelling ^10^Be concentrations. Across the 300-m Hartle Loup platform profile (shown in Fig. 1) under 58 scenarios of changing cliff retreat rates (colour scale) and 4 scenarios of foreshore down-wearing: **a** zero-surface down-wearing, **b** profile-parallel coastal erosion, **c** platform widening and **d** empirically based model of platform down-wearing. Points represent measured ^10^Be concentrations corrected for geological inheritance (concentration at #0 subtracted from raw measurements) ± AMS measurement error and background- and inheritance uncertainties propagating in quadrature (Supplementary Note 1).

Figure 3 illustrates ^10^Be concentrations modelled using all combinations of cliff retreat rates and trends (58 scenarios) and shore platform erosion (four scenarios). The acceleration scenarios only explain the formation of the landward portion of the shore platform over the last 7 kyr. The saw-toothed profile of ^10^Be concentrations matches predictions related to the presence of near-horizontal sedimentary beds eroding by step back-wearing^13^. This is apparent in both steady and decelerating cliff retreat rate scenarios within the zero-surface down-wearing, profile-parallel coastal erosion and platform widening (Fig. 3a–c). The latter model reveals a subtle “hump” in concentrations that was observed and modelled in previous studies^13,15,16^. Only in the empirically based models do different cliff retreat scenarios result in similar ^10^Be concentrations (Fig. 3d), which underpredict the measured concentrations. Model verification against the three criteria showed that all acceleration scenarios, scenarios with slow (≤4 cm yr^−1^) steady retreat rates and the slowest deceleration scenario were not able to produce a 300-m-wide platform over the 7-kyr period. This means that 112 scenarios (48%) did not fulfil criterion 1. Of the remaining set, 41 scenarios (18% total) fulfilled statistical similarity criterion 2 with NRMSD ranging from 0.8 to 0.19. Although 113 scenarios (49%) fulfilled criterion 3, these were mostly the acceleration scenarios and those assuming slow steady rates, and so only six successful scenarios overlapped with those that fulfilled criterion 1. Of these, only one scenario fulfilled all three criteria (see Methods and Supplementary Figs. 1–4).

### Long-term coastal erosion rates

The only modelled scenario that fulfilled all three criteria (NRMSD = 0.14) was for a steady cliff retreat rate of 4.5 ± 0.63 cm yr^−1^ and a profile-parallel erosion model with down-wearing of 0.314 ± 0.044 mm yr^−1^ adjusted with RSL rise (Eq. (2) in “Methods”)^16,21^ to maintain a constant platform slope of ~0.4° (Fig. 2b).

We calculated the long-term back-wearing rate of the steps at the termini of beds B1 and B2 after selecting the most likely cliff retreat and platform down-wearing scenarios based on the difference between measured and modelled ^10^Be concentrations ≥231 m from the cliff (see “Methods”). The long-term back-wearing rate of the 0.80-m step at the terminus of B1 was modelled as 0.60 ± 0.35 cm yr^−1^. Using a similar approach, the 0.75-m step at the terminus of B2 has been retreating at the average rate of 1.51 ± 0.44 cm yr^−1^.

## Discussion

The key findings of our study are: (i) over long timescales, cliff retreat rate has been relatively steady, (ii) the coastal profile has retained a stable shape while migrating landwards and (iii) steps delimiting beds at the foreshore have been back-wearing at rates less than half that of cliff retreat.

Our data demonstrate that reconstructed rates of cliff retreat at our site have been steady despite ~5.8-m RSL rise over the last 7 kyr^21,22^. Although a decreasing rate of RSL rise was considered within our model to derive exposure ages from ^10^Be concentrations, no direct link with Holocene cliff erosion rate could be identified. ^10^Be concentrations gradually increase seaward, suggesting that the Hartle Loup platform cannot be a feature inherited from the last interglacial (130–118 kyr BP) (cf. Agar^37^). The shore platform must therefore have formed entirely within the last 7 kyr, as also suggested for sites further south in the English Channel^15,16^. The potential impact of the Medieval Climatic Anomaly (MCA) and the Little Ice Age (LIA)^25–27^, and the recent post-industrial acceleration since early nineteenth century^38,39^ cannot be resolved from our data as only two sites (#1–2, 31–50 m) overlap with portions of foreshore likely to have been influenced by these climatic/industrial events (Fig. 2b). At our study site, the rate of RSL rise does not therefore appear to have controlled the long-term rate of coastal retreat, and it is not possible to infer a direct correlation between rates of rocky coast erosion and RSL and/or rates of RSL change. Unlike the sites studied by Hurst et al.^16^, rates of erosion at our study location are highly unlikely to have been influenced by changes in beach cover due to the exposed headland morphology that prohibits beach formation. This difference in observations may be due to local spatial variability along-coast. RSL rise controls local morphodynamic processes that, in turn, influence local rates of erosion and retreat^16^, the efficacy of which remains controlled locally by cliff and foreshore rock mass resistance^40,41^. It is therefore likely that erosion in response to changing RSL might be highly variable along even short (<1 km) stretches of coastline^20^, and this must be considered when selecting sites for studies such as this. This variability also mirrors apparent differences in the sensitivity of erosion to human activity observed across the English Channel^15,16^. Future predictions of coastline change under anticipated RSL rise^1^ should therefore be considered as locally contingent; other factors such as changes in nearshore wave climate and the local rock resistance of the coast may be more important in driving erosion^8,42,43^.

The long-term cliff retreat rate is similar to the average global retreat of the hard rock to medium-strength rock coastlines of 2.9–10 cm yr^−1^ reported on the basis of 406 individual site observations^44^. The rates fall into the 2.7 ± 2.9 cm yr^−1^ range calculated for the nearby cliffs from 7 years of high-resolution monitoring using terrestrial laser scanning^30^. However, the possibility of confidently extrapolating contemporary erosion rates to infer longer-term (10^2^–10^3^ yr) cliff dynamics should be further addressed. For example, Regard et al.^15^ found that retreat rates of S English Channel cliffs have been of the same order of magnitude over 6 kyr and 30 yr. Our data also complement much shorter timescale observations, such as Williams et al.^45^ who established that over monitoring windows of ca. 12 h, the form of the volume-frequency relationship of cliff retreat via rockfall appears to remain near-steady, irrespective of how often erosion is measured, suggesting that an incremental mode of erosion is dominant.

Despite steady long-term average cliff retreat rates, we observe two apparently anomalous observations along the non-stepped section of the foreshore. Firstly, the ^10^Be concentrations of sample #3 (62 m) are notably lower than those of #4 (72 m). Sample #3 is located 62 m from the cliff, a distance equivalent to 1,378 yr BP of the steady retreat at 4.5 cm yr^−1^. As no major sea-level event is known to have occurred around this time (Fig. 2b), we interpret the lower-than-predicted concentrations, and hence temporarily higher retreat, to be most likely related to intrinsic factors such as local change in rock resistance, a non-coastline-normal erosion of a headland or the episodic nature of erosion over the short term^10^. The slower retreat might have run in fact from 1,844 yr BP (#5, 83 m). In contrast, the relatively low ^10^Be concentration in sample #10 (213 m, ~4,733 yr BP) may be due to non-coastline-normal stripping of intra-bed sandstone layers, given the proximity of this site to B1/B2 step. This should be addressed further by more dense sampling both across- and along-shore.

The shore platform erosion model that best fits our measured concentrations assumes surface down-wearing proportional to the combination of cliff retreat and RSL rise (Eq. (2) in Methods), where the profile of the shore platform slope remains unchanged^3^. Importantly, this model outperforms those that consider negligible foreshore erosion, empirically based spatially variable erosion or a platform-gradient change (platform widening). This successful form of the model was also previously used in cosmogenic studies of rocky coast erosion rates^15,16^. Our results confirm that foreshore erosion is an important consideration in developing models to explain cumulative isotope concentrations (cf. Choi et al.^14^), and that long-term and wide-area monitoring of foreshore erosion is vital in explaining local variations in the erosion rates.

Our calculated long-term platform down-wearing rate of 0.314 ± 0.044 mm yr^−1^ is one order of magnitude lower than that measured with a micro-erosion meter^31^, which suggests that short-term erosion rates derived from point measurements should not be extrapolated in space and time, at least at some locations^19^. The long-term erosion rate is comparable to the 0.222 ± 0.122 mm yr^−1^ calculated for the sandstone sites of the same platform from one year of monitoring^32^. However, the empirical model that predicts the spatial distribution of erosion based on this study, where down-wearing rates were higher further from the seaward edge and at locations with higher tidal duration, is not able to reproduce observed ^10^Be concentrations. As such, the spatial and temporal differences in erosion rates across the platform and their adjustment to a changing shore platform geometry must average out over the centennial to millennial timescales considered here. This suggests that in future, a process-based forward model of shore platform formation^7^ should be coupled with cosmogenic exposure dating to explore a wide range of morphologic situations that result in specific across-shore ^10^Be distributions.

This study is the first to calculate the long-term back-wearing rate of step features on the foreshore. The impact of this process on the distribution of cosmogenic ^10^Be is seen in the saw-toothed distribution in Fig. 2b, which has previously only been conceptually demonstrated^13^. Although block detachment is often observed on shore platforms^18,19^, very few studies have reported rates of step back-wearing; on the chalk platforms of the English Channel, Dornbusch and Robinson^46^ reported rates of 1.25–18.1 cm yr^−1^, and broadly comparable rates were reported by Regard et al.^47^ (1.8–3.0 cm yr^−1^). The spatial and temporal variability of such erosion rates, the relatively short duration of the monitoring period and the low precision of monitoring (~10 cm) make it difficult to use such data to understand longer-term step dynamics. Although based on only two steps, the rates of 0.60 ± 0.35 cm yr^−1^ and 1.55 ± 0.44 cm yr^−1^ obtained here reflect long-term (centuries to millennia) change, and so offer a more accurate basis for extrapolation. Regard et al.^47^ observed that at the decadal scale, steps retreat slower than the cliff, and that they contribute to 20–100% of foreshore erosion. Buchanan et al.^48^ suggested that block detachment may dominate foreshore erosion in S Wales. Similarly, here we show that over long timescales, step back-wearing is ~25% of cliff erosion (0.60 ± 0.35 cm yr^−1^ and 1.55 ± 0.44 cm yr^−1^ vs. 4.5 ± 0.63 cm yr^−1^). This may be due to the wave energy distribution across the foreshore^7,20,49^ or different responses to the various drivers of cliff and step erosion^18,30^. We estimate the contribution of step back-wearing as 14.6% of the total volumetric foreshore erosion with the remainder 85.4% being due to surface down-wearing. This suggests that both the micro- and mesoscale foreshore erosion processes should be included in numerical modelling^5–7^ to more accurately reflect shore platform dynamics that, in turn, allows us to predict future change.

We have presented exposure dating using cosmogenic ^10^Be concentrations across the macro-tidal 300-m Hartle Loup shore platform in North Yorkshire, UK. The results show that the cliff has been retreating at a steady rate of 4.5 ± 0.63 cm yr^−1^ cutting the present foreshore entirely within the last 7 kyr. The shore platform is not inherited from previous sea-level high stands. The retreat of the coast has been insensitive to rates of RSL change. The long-term cliff retreat rate modelled from exposure ages is consistent with high-resolution monitoring of contemporary cliff retreat, not only in terms of average rate, but also in terms of longer-term predictions of retreat from modelling contemporary cliff rockfall magnitude–frequency, and hence erosion^40,41^.

Over millennial timescales, the down-wearing of the shore platform has been dictated by the rate of cliff retreat and RSL rise, maintaining constant slope of 0.4°. The average surface down-wearing rate of 0.314 ± 0.044 mm yr^−1^ again is comparable to rates obtained from 1-year high-resolution monitoring, but an order of magnitude lower than nearby estimates derived using a micro-erosion meter. This study is the first to measure long-term rates of back-wearing of steps upon the foreshore at 0.60 ± 0.35 and 1.55 ± 0.44 cm yr^−1^, which contribute to 14.6% of foreshore erosion. Combined, these new longer-term estimates of the various components of rock coast erosion are essential for forward modelling the likely nature of future change.

## Methods

### Sampling

We collected ~2-kg rock samples at 20 locations along the 300-m profile and at one unexposed location at the bottom of the cliff (Fig. 1b) to account for deep muon production, referred to as geological inheritance^16^. The specific locations were selected to calculate the average cliff retreat rate in the Holocene and establish whether the coast is inherited from the interglacial, and separate the importance of surface down-wearing and step back-wearing (block removal) in foreshore erosion over long timescales (sampling at 10-m intervals and at tops and bottoms of steps >230 m from the cliff) (Fig. 1b).

### Laboratory methods

We separated beryllium isotopes from sandstone samples in the CosmIC laboratory at Imperial College London using standard procedures^40–52^, with a minor adjustment to permit capture and purification of quartz grains ranging in size from 53 to 106 μm. To do so, we (i) wet-sieved the crushed and milled material, and (ii) performed two 9-h rounds of HF etching in a heated 90 °C ultrasonic tank using 15 g of sample/L 0.5% HF/HNO_3_. The purity of the final quartz separates was verified using an Agilent 5100 SVDV ICP-OES. The concentrations of aluminium in quartz were between 185 and 280 ppm for the 21 samples. The AMS ^10^Be/^9^Be ratios were measured using 6 MV Sirius tandem accelerator at the Centre for Accelerator Science at the Australian Nuclear Science and Technology Organisation^53^ and normalised to the KN-5-2 standard with an assumed ratio of 8.558 × 10^−12^ ^54^. The ratios were converted into ^10^Be concentrations and corrected for background and geological inheritance with the error propagated in quadrature (Supplementary Note 1; Supplementary Data 1; Supplementary Tables 1 and 2).

### Modelling

We assumed that (i) the cliff retains a constant morphology; (ii) after initial exposure, the shore platform surface is never reburied; (iii) there is zero shielding due to beach cover. This assumption is based on the absence of beaches in historical maps and photographs, at present, and more generally at the headland sections of rocky coasts, and (iv) there have been no long-term trends in changing wave climate or tidal regime over the period studied.

If a portion of the platform had been inherited from previous sea-level high stands (interglacials), there would be an abrupt increase in the concentrations at some across-shore location that would delimit currently formed and inherited sections due to difference of >100 kyr of exposure^15^. As we did not observe such an increase (Supplementary Table 2), we assumed that the present platform is entirely formed after 7 kyr BP, when RSL reached ca. −5.8 m above ordinance datum (AOD), and hence when erosion of the modern coast could commence^21,22^

1 $$\left[{{\;}^{{\it{10}}}Be} \right] = P\;t_{\exp }\,S_{{\mathrm{topo}}}\,S_{\mathrm{w}}\,S_{{\mathrm{gm}}}\,S_{{\mathrm{er}}},$$

where *[*^*10*^*Be]* (atoms g^−1^) is the total concentration of ^10^Be, *P* = 4.009 atoms g^−1^ yr^−1^ is the reference sea-level high-latitude (SLHL) ^10^Be production rate at the surface from the unshielded flux^35^, *t*_exp_ (yr) is the time of exposure, *S*_topo_ is the spatially explicit topographic shielding^36^, *S*_w_ is the water shielding that depends on the sea-level change and tidal duration distribution^33^, *S*_gm_ is the geomagnetic scalar^35^ and *S*_er_ is the platform erosion scalar. The latter scalar has been defined in this study. It accounts for the fact that rock samples exposed at the surface at present, in the past have been at some depth that can be calculated from platform erosion rates, and so their production rate was lower than *P*. The scalars are expressed relative to 1, where 1 is the non-shielded value. *S*_topo_, *S*_w_ and *S*_er_ adopt values ≤1, depending on the importance of the factor (1 means no impact), while *S*_gm_ can adopt values higher or lower than 1 and is relative to the mean long-term surface production rate.

Due to the headland location of the sampling profile (Fig. 1b) and the complex geometry of the coastline, we calculated the topographic shielding based on a LiDAR-derived Digital Elevation Model (spatial resolution 0.2 m, 2016) using the model of Mudd et al.^36^ under the assumption of α = 5° and φ = 5°. We used the water-shielding model of Lal^33^ that combines the effects of tidal duration distribution (data obtained from https://www.bodc.ac.uk/) with the RSL change^21^. We accounted for geomagnetic and solar modulation effects on the flux of cosmic-ray particles through time using the model of Lifton et al.^35^

We considered 58 scenarios of cliff retreat rates, of which 20 assumed a steady rate, 19 a linear acceleration and 19 a linear deceleration. For the steady model, we considered rates of 0.5–10 cm yr^−1^ at 0.5 cm yr^−1^ intervals. For the scenarios where the rate changed over time, we used the present rate of 2.5 cm yr^−1^ ^30^, made the rate 2–20 times lower (acceleration scenarios) or higher (deceleration scenarios) at 7 kyr BP and adjusted the intermediate rates linearly. We used these rates to calculate across-shore exposure ages for the 58 scenarios that in turn allowed the calculation of the cumulative *S*_gm_ and *S*_topo_ (Supplementary Fig. 1).

The structure of the Hartle Loup platform (layering, bedding, jointing and presence of steps) suggests that the platform erosion scalar, *S*_er_ results from the sub-mm to cm-scale erosion via abrasion and detachment of rock^31,32^, and the m-scale block removal^18^. For simplicity, we refer to these processes at the respective spatial scales: down-wearing and step back-wearing, and the respective platform erosion scalars are referred to as *S*_er_down_ and *S*_er_step_. Based on the literature, we explored four different models of long-term platform evolution that would result in different across-shore distribution of *S*_er_down_:

1. The zero-surface down-wearing model (*S*_er_down_ = 1) that ignores foreshore erosion. This model was used by Choi et al.^14^.
2. The profile-parallel erosion model in which the down-wearing is proportional to cliff retreat rate and the coastal cross-profile (notably the shore platform gradient) retains a constant shape^3^. The model takes into account the RSL change and so the specific platform erosion rate for each year can be calculated as
  2 $$Ero = r\;\tan \alpha - {\mathrm{RSL}},$$
  where *Ero* (mm yr^−1^) is the platform erosion rate, *r* (mm yr^−1^) is the cliff retreat rate, *α* (°) is the platform gradient and RSL (mm yr^−1^) is the RSL rise^3^. This model was used by Regard et al.^15^ and Hurst et al.^16^
3. The platform-widening model in which the seaward edge located below the wave base is laterally and vertically stable, and the platform becomes wider and flatter as the cliff retreats^2,13^.
4. The empirical model developed for the Hartle Loup platform^32^ in which the erosion rate is higher further from the seaward edge and at locations of more frequent tidal induration, such that
  3 $$\\ {\it{Ero}} = - {{2}}{{.01}} + {{0}}{{.01}}\;{\it{Sea}} + {{0}}{{.60}}\;{\it{Tid}},$$
  where *Ero* (mm yr^−1^) is the erosion rate, *Sea* (m) is the distance from the seaward edge of the platform and *Tid* (%) is the tidal duration derived from 2006 to 2010 hourly data of NERC British Oceanographic Data Centre (https://www.bodc.ac.uk/)^32^.

We explored the scenarios back through time, which means that after calculating point down-wearing for a year *t*_n_, we adjusted the point elevation in year *t*_*n*–1_, which could then be used to calculate *S*_w(*n*–1)_ and at a point down-wearing in *t*_*n*–1_, and so on. For all scenarios, we set a boundary condition that at any point, platform elevation could not exceed the highest astronomical tide (HAT = 3.2 m) relative to the RSL at that point. Supplementary Figs. 2 and 3 show total across-shore distributions of *S*_w_ and *S*_er_ for different scenarios of foreshore down-wearing.

We selected the most likely combinations of cliff retreat (58 scenarios) and foreshore down-wearing (4 scenarios) according to their match with measured ^10^Be concentrations corrected for the geological inheritance^16^ using three criteria for fulfilment: (i) the whole profile had to be exposed ≤7 kyr BP, (ii) the normalised root-mean-square deviation (NRMSD) between measured and modelled concentrations of non-stepped section of the profile (#1–12) had to be <0.2 and (iii) all modelled concentrations from the stepped section of the profile (#13–20) had to be ≥ measured concentrations.

Coupling Eq. (1) with the across-shore distribution of exposure ages and shielding/scaling factors for 58 scenarios of cliff retreat and 4 scenarios of shore platform down-wearing (Supplementary Figs. 1–3) allowed the modelling of ^10^Be concentrations along the predefined topographic profile (Fig. 3). The concentrations predicted by 232 (58 × 4) scenarios were then verified in terms of fulfilment of the three criteria at 20 sampling sites. In total, 28 (48%) of 58 scenarios of cliff retreat did not explain formation of the whole 300-m profile within the last 7 kyr and so did not fulfil criterion 1. These are all acceleration scenarios, scenarios of steady retreat ≤4 cm yr^−1^ and the slowest deceleration scenario (in which the retreat rate was double that at present at 7 kyr). In total, 41 scenarios (18%) fulfilled the statistically objective criterion 2. These are a subset of the steady cliff retreat scenarios of rates between 4.5 and 8.5 cm yr^−1^ and deceleration scenarios with zero foreshore down-wearing (11 scenarios), profile-parallel coastal erosion (5 scenarios) and platform widening (7 scenarios) (Supplementary Table 3). All acceleration scenarios and lower steady-rate scenarios predicted equal or higher concentrations at the stepped (seaward) section of the foreshore (fulfilment of criterion 3). The only scenario that fulfilled the three criteria assumes the steady cliff retreat rate of 4.5 cm yr^−1^ and profile-parallel coastal erosion (Supplementary Fig. 4). The NRMSD = 0.14 (criterion 2) is assumed to represent uncertainty of both the cliff retreat rate and the platform down-wearing, as reported in the main paper.

The platform erosion scalar, *S*_er_ was assumed to result from the combination of platform down-wearing and step back-wearing, with the respective contribution referred to as *S*_er_down_ and *S*_er_step_. The steps are located at the seaward 75 m of the foreshore profile, and so we first found the most likely scenario (or the best-fit model) of long-term platform down-wearing for the landward 231 m and extrapolated it to the seaward 70 m (#13–20). We calculated *S*_er_step_, notably applicable only to the seaward 70 m due to the location of steps, by dividing the inheritance-corrected ^10^Be concentrations (Supplementary Table 2) by the concentrations predicted by the best-fit model.

We used the results to calculate the long-term step back-wearing rates. Knowing the exposure times of the sampling locations allows the calculation of exposure time from the rock beds B1 (top step) or B2 (bottom step). This is based on the relative contribution of *S*_er_step_ < 1 for the period of time when the location was exposed from under the cliff, but the step had not receded yet, and *S*_er_step_ = 1 for the period when the sample was exposed from under the bed (Supplementary Table 4).

The difference between measured and modelled ^10^Be concentrations reflects the platform erosion scalar due to the step back-wearing contribution, *S*_er_step_. The scalar adopts the lowest values immediately at the foot of the steps and gradually increases seaward. The top step (at 232 m from the cliff) is 0.80 m high, and the bottom one (at 271 m) is 0.75 m high. The step heights also reflect thickness of the sandstone beds B1 and B2, respectively. For a single year before a location experienced step back-wearing, *S*_er_step_ equals 0.54 and 0.56 for the respective steps (as calculated from the step height or the bed thickness) and 0.30 if both beds were over a site located on the bed B3^34^. For example, step retreat rate at site #13 is calculated as follows. First, the total *S*_er_step_ is calculated as measured ^10^Be concentrations divided by the modelled ones (7640/10,986 = 0.70) (Supplementary Fig. 5). Then, the time when the top step was at #13 (236 m) is calculated as

4 $$	\, \left( {\left( {S_{{{{\rm{er}}\_{\rm{step}}\_{\rm{total}}}}} - S_{{{{\rm{er}}\_{\rm{step}}\_{\rm{1yr}}}}}} \right) \times t_{{\mathrm{exp}}}} \right)/\left( {1 - S_{{{{\rm{er}}\_{\rm{step}}\_{\rm{1yr}}}}}} \right)\\ 	\,= \left( {\left( {0.70 - 0.54} \right) \times 5246} \right)/\left( {1 - 0.54} \right),$$

Finally, the step retreat rate is quantified by dividing the distance from the step by the time the top step was at #13 and multiplied by 100 (4/1773 × 100 = 0.23).

The average step back-wearing rates are 0.60 ± 0.35 cm yr^−1^ for the top (i.e. landward) step and 1.51 ± 0.44 cm yr^−1^ for the bottom (i.e. seaward) step averaged from the rates calculated for individual sites (mean ± standard deviation). We treated the rate obtained at site #17 (274 m) as an outlier because of an anomalously high step back-wearing rate of 8.57 cm yr^−1^. Episodic character of block detachment from the foreshore^19^ may make this site located 3 m from the step unsuitable to derive the long-term erosion rates^9,10^.

We calculated the contribution of surface down-wearing and step back-wearing to the total volumetric foreshore erosion by multiplying obtained rates by the relevant platform metric. The relative contribution of down-wearing equals 0.314 mm yr^−1^ times 300 m, while the contribution of step back-wearing equals 0.60 cm yr^−1^ times 0.8 m plus 1.55 cm yr^−1^ times 0.75 m. Hence, we estimate that step back-wearing makes up 14.6% of the foreshore erosion.

## Supplementary information

Supplementary Information

Description of Additional Supplementary Files

Supplementary Data 1

Supplementary Data 2

## Data Availability

We confirm that all new data associated with this paper are supplied in the associated supplementary files. Supplementary Data 1 and the Supplementary Information file contain details on the calculation of ^10^Be concentrations in samples and AMS uncertainties, and Supplementary Data 2 contains information on the modelling.

## Code availability

A MATLAB code and all input matrices used to explore and visualise the scenarios of cliff retreat and shore platform down-wearing are made available in Supplementary Data 2.
