# Publisher Correction: Cosmogenic exposure dating reveals limited long-term variability in erosion of a rocky coastline

**DOI:** 10.1038/s41467-020-18294-y

**Published:** 2020-08-27

**Authors:** Zuzanna M. Swirad, Nick J. Rosser, Matthew J. Brain, Dylan H. Rood, Martin D. Hurst, Klaus M. Wilcken, John Barlow

**Affiliations:** 1 Department of Geography, Durham University, South Road, Durham, DH1 3LE UK; 2 Scripps Institution of Oceanography, University of California San Diego, 9500 Gilman Drive, La Jolla, CA 92093 USA; 3 Department of Earth Science and Engineering, Imperial College London, South Kensington Campus, London, SW7 2AZ UK; 4 School of Geographical and Earth Sciences, University of Glasgow, University Avenue, Glasgow, G12 8QQ UK; 5 Australian Nuclear Science and Technology Organization, New Illawarra Road, Lucas Heights, NSW 2234 Australia; 6 Department of Geography, University of Sussex, Brighton, BN1 9QJ UK

**Keywords:** Ocean sciences, Physical oceanography, Geochemistry, Geomorphology

Correction to: *Nature Communications* 10.1038/s41467-020-17611-9, published online 30 July 2020.

The original version of this Article contained an error in the Abstract, in which the word ‘erosive’ was misspelled ‘eros0ive’. This has now been corrected in the PDF and HTML versions of the Article.

