# Supplementary material for: Cosmogenic exposure dating reveals limited long-term variability in erosion of a rocky coastline
Source: Nat Commun. 2020 Jul 30;11:3804. doi: 10.1038/s41467-020-17611-9 (PMC7393086; doi:10.1038/s41467-020-17611-9)
Supplement: Supplementary file 1 — Supplementary Information [file 41467_2020_17611_MOESM1_ESM.pdf]

SUPPLEMENTARY INFORMATION

Cosmogenic exposure dating reveals limited long-term variability in erosion of a rocky coastline

Swirad et al.

SUPPLEMENTARY NOTES

Supplementary Note 1: Calculation of ^{10}Be concentrations in samples and AMS uncertainties

Before quarts dissolution, ~0.334 g of the UMV 17-1 ^{9}Be carrier (759 ± 9 ppm) was added to the samples in order to calculate the AMS $^{10}Be/^{9}Be$ ratio[1]. The procedure was performed in three batches, each having a blank (#BLK1-3), which did not contain quartz (Supplementary Table 1).

The AMS $^{10}Be/^{9}Be$ ratio was measured ten times for each sample and the measurement error was calculated by comparing the Poisson counting statistics to the standard deviation between these measurements. The measurement error was expressed by absolute ratio values and re-calculated to percentage so that it could be used to calculate uncertainty of calculated ^{10}Be concentrations. The quantity of ^{10}Be atoms was calculated by multiplying the AMS $^{10}Be/^{9}Be$ ratio by the quantity of ^{9}Be atoms added with the carrier. The background quantity of ^{10}Be atoms was averaged from the three blanks (11,333 atoms) and it was subtracted from the total quantity of ^{10}Be atoms in each sample. The standard deviation of the total number of ^{10}Be atoms in the blank (6,169 atoms) was used to estimate the uncertainty on the background. The uncertainties in the sample measurement error and background were propagated in quadrature. The background-corrected ^{10}Be concentrations and uncertainties were calculated by dividing the background-corrected amount of ^{10}Be by the quartz mass (Supplementary Data 1).

A concentration of 1,304 ± 268 atoms g^{-1} – the background-corrected concentration of the unexposed sample (#0) – was assumed to represent the geological inheritance[2], and hence was treated as the baseline above which greater concentrations result directly from a function of near-surface production, exposure time, topographic and water shielding, and foreshore erosion. When comparing measured and modelled ^{10}Be concentrations, 1,304 atoms g^{-1} was subtracted from the measured ^{10}Be concentrations to represent the inheritance-corrected ^{10}Be concentrations. The uncertainties for the exposed and unexposed samples were propagated in quadrature (Supplementary Table 2).

SUPPLEMENTARY TABLES

Supplementary Table 1. Details on the ^{9}Be carrier added to the samples before quartz dissolution.

Sample #	^{9}Be carrier mass (g)	^{9}Be carrier concentration (μg g^{-1})	Quantity of ^{9}Be (atoms) (×10^{19})
0	0.3406	759	1.72745
1	0.3394	759	1.72137
2	0.3383	759	1.71579
3	0.339	759	1.71934
4	0.3416	759	1.73252
5	0.3412	759	1.7305
6	0.3379	759	1.71376
7	0.3398	759	1.72339
8	0.344	759	1.7447
9	0.3395	759	1.72187
10	0.3304	759	1.67572
11	0.3409	759	1.72897
12	0.3382	759	1.71528
13	0.3419	759	1.73405
14	0.3374	759	1.71122
15	0.3403	759	1.72593
16	0.3404	759	1.72644
17	0.3361	759	1.70463
18	0.3358	759	1.70311
19	0.34	759	1.72441
20	0.3366	759	1.70716
BLK1	0.3396	759	1.72238
BLK2	0.3405	759	1.72694
BLK3	0.3361	759	1.70463

Supplementary Table 2. The correction of measured ^{10}Be concentrations with the geological inheritance in order to compare the measured concentrations with model predictions; *AMS measurement and background errors only. The data from this table are visually presented in Fig. 3 of the main document.

Sample #	Distance from the cliff (m)	Background-corrected ^{10}Be concentration (atoms g^{-1})	Inheritance-corrected ^{10}Be concentration (atoms g^{-1})	Inheritance-corrected uncertainty (atoms g^{-1})
0	n/a	1,304	n/a	268*
1	31	2,700	1,396	421
2	50	3,197	1,893	404
3	62	2,643	1,339	402
4	72	3,531	2,227	403
5	83	4,866	3,562	439
6	93	5,492	4,188	487
7	101	6,397	5,093	695
8	150	9,209	7,905	600
9	198	10,457	9,153	583
10	213	10,174	8,870	581
11	220	12,001	10,697	603
12	231	11,559	10,255	522
13	236	8,944	7,640	542
14	248	11,019	9,715	1229
15	260	10,769	9,465	636
16	267	13,630	12,326	781
17	274	7,498	6,194	590
18	280	8,454	7,150	789
19	288	10,338	9,034	648
20	299	12,700	11,396	596

Supplementary Table 3. Normalised root-mean-square deviation (NRMSD) between ^{10}Be concentrations at 12 landward points (#1-12) measured and modelled with the 232 scenarios of the long-term cliff retreat and shore platform down-wearing rates. Scenarios that do not fulfil the criterion 1 of shore platform formation within the last 7 kyr do not have a value. Scenarios that fulfil the criterion 2 of NRMSD < 0.2 are in bold.

	n	Zero surface down-wearing	Profile-parallel coastal erosion	Platform widening	Empirical model of platform erosion
Linear deceleration (*n* times higher 7 kyr BP)	20	0.22	0.29	0.27	0.34
	19	0.21	0.28	0.26	0.34
	18	0.21	0.27	0.25	0.33
	17	0.20	0.27	0.25	0.33
	16	**0.19**	0.26	0.24	0.33
	15	**0.18**	0.25	0.23	0.32
	14	**0.18**	0.24	0.23	0.32
	13	**0.17**	0.23	0.22	0.32
	12	**0.16**	0.22	0.21	0.31
	11	**0.15**	0.21	0.20	0.31
	10	**0.15**	0.20	**0.19**	0.31
	9	**0.15**	**0.19**	**0.18**	0.30
	8	**0.15**	**0.18**	**0.18**	0.30
	7	**0.16**	**0.17**	**0.17**	0.29
	6	**0.19**	**0.16**	**0.17**	0.29
	5	0.23	**0.16**	**0.17**	0.29
	4	0.30	0.20	**0.18**	0.28
	3	0.41	0.28	0.21	0.28
	2				
Steady retreat rate (*n* cm yr^{-1})	0.5				
	1				
	1.5				
	2				
	2.5				
	3				
	3.5				
	4				
	4.5	0.30	**0.14**	**0.12**	0.28
	5	0.20	**0.09**	**0.11**	0.28
	5.5	**0.13**	**0.11**	**0.12**	0.29
	6	**0.09**	**0.14**	**0.14**	0.29
	6.5	**0.08**	**0.18**	**0.17**	0.30
	7	**0.10**	0.21	**0.19**	0.31
	7.5	**0.12**	0.23	0.21	0.31
	8	**0.15**	0.26	0.23	0.32
	8.5	**0.17**	0.28	0.25	0.33
	9	0.20	0.30	0.27	0.34
	9.5	0.22	0.31	0.29	0.34
	10	0.23	0.33	0.30	0.35
Linear acceleration (*n* times lower 7 kyr BP)	2				
	3				
	4				
	5				
	6				
	7				
	8				
	9				
	10				
	11				
	12				
	13				
	14				
	15				
	16				
	17				
	18				
	19				
	20				

Supplementary Table 4. Calculation of step back-wearing rate at the seaward portion of the Hartle Loup platform. The step retreat rates at sites #13-16 (236-267 m) were used to calculate the top step back-wearing rate, while the rates at sites #18-20 (280-299 m) were used to derive the bottom step back-wearing rate. Site #17 (274 m; erosion rate marked with *) was considered an outlier and was not used to derive the long-term step back-wearing rates.

Site	Distance from the cliff (m)	Site exposure age (yr)	Total S_{er_step}	Distance from the top step (m)	Distance from the bottom step (m)	1 yr S_{er_step} (top step)	1 yr S_{er_step} (bottom step)	Time of exposure form under the bed (yr)	Time the top step was at #17-20 (yr BP)	Time underneath both beds (yr)	Step retreat rate (cm yr^{-1})
13	236	5,246	0.70	4	n/a	0.54	n/a	1,773	n/a	n/a	0.23
14	248	5,513	0.85	16	n/a	0.54	n/a	3,717	n/a	n/a	0.43
15	260	5,779	0.76	28	n/a	0.54	n/a	2,772	n/a	n/a	1.01
16	267	5,935	0.90	35	n/a	0.54	n/a	4,676	n/a	n/a	0.75
										Average top step back-wearing rate	0.60 ± 0.35
17	274	6,090	0.55	42	3	0.30	0.56	35	5,764	326	8.57*
18	280	6,224	0.60	48	9	0.30	0.56	568	6,470	n/a	1.59
19	288	6,401	0.62	56	17	0.30	0.56	891	7,411	n/a	1.91
20	299	6,646	0.74	67	28	0.30	0.56	2,696	8,705	n/a	1.04
										Average bottom step back-wearing rate	1.51 ± 0.44

SUPPLEMENTARY FIGURES

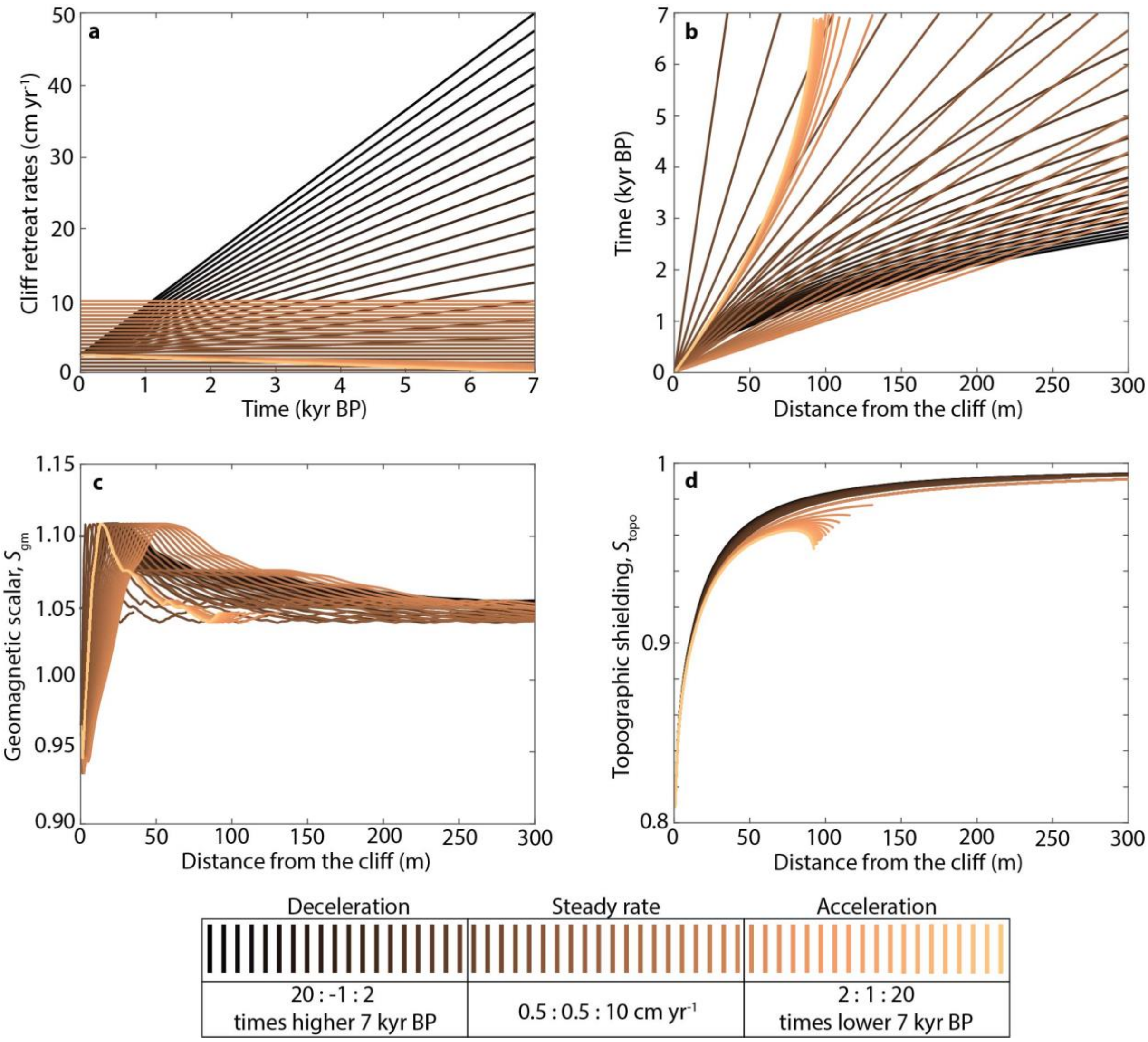

Supplementary Figure 1. Results of modelling exposure ages and shielding/scaling factors across the 300 m profile (Fig. 1 in the main document) under 58 scenarios of cliff retreat: a) cliff retreat rate; b) exposure age; c) geomagnetic scalar; d) topographic shielding. Specific cliff retreat scenarios are consistently coloured and explained in the bottom colour-scheme plot.

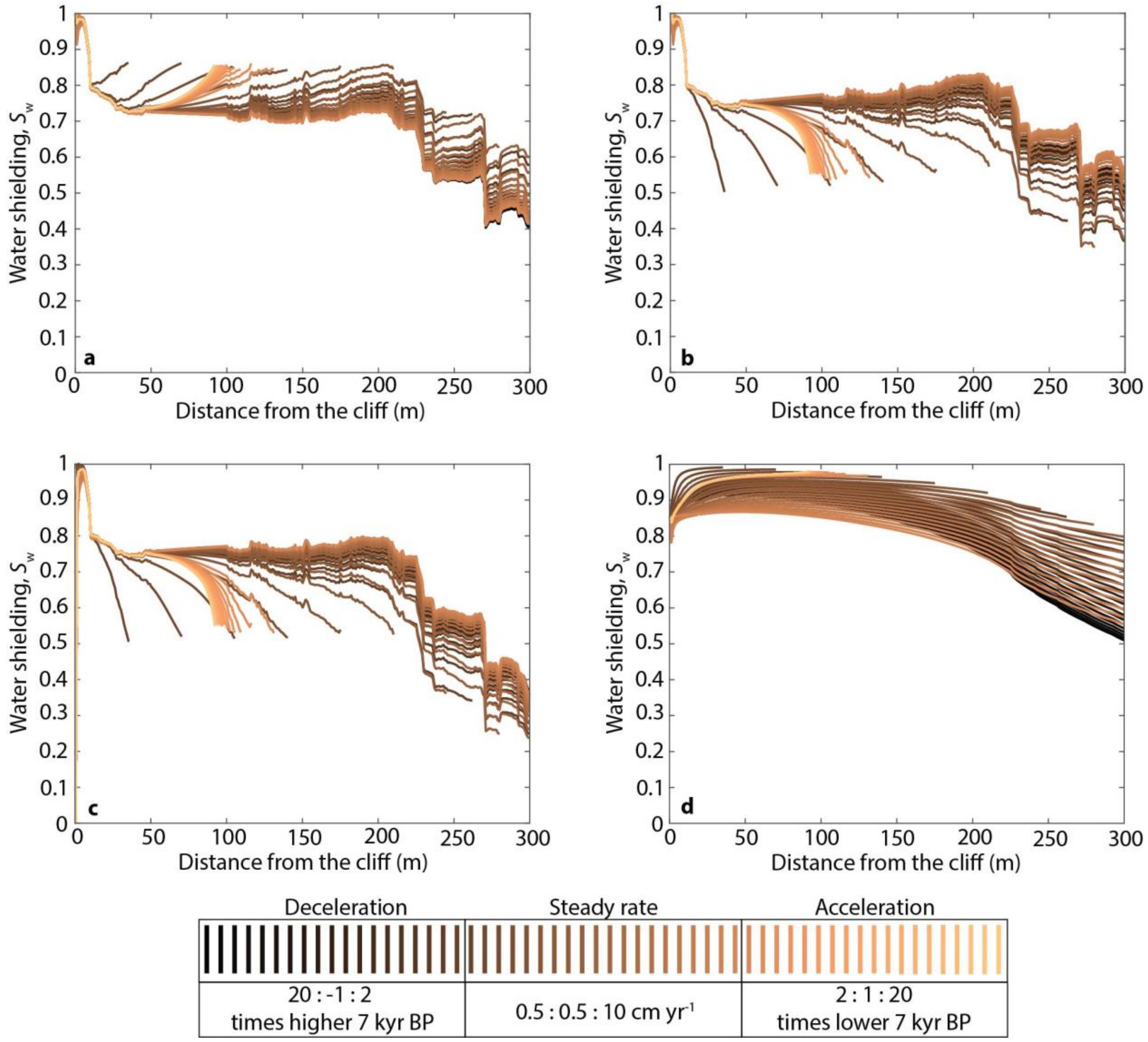

Supplementary Figure 2. Results of modelling total water shielding across the 300 m profile (Fig. 1 in the main document) under 58 scenarios of cliff retreat and: a) zero platform down-wearing; b) profile-parallel erosion model; c) platform widening; d) empirically-based model of platform down-wearing. Specific cliff retreat scenarios are consistently coloured and explained in the bottom colour-scheme plot.

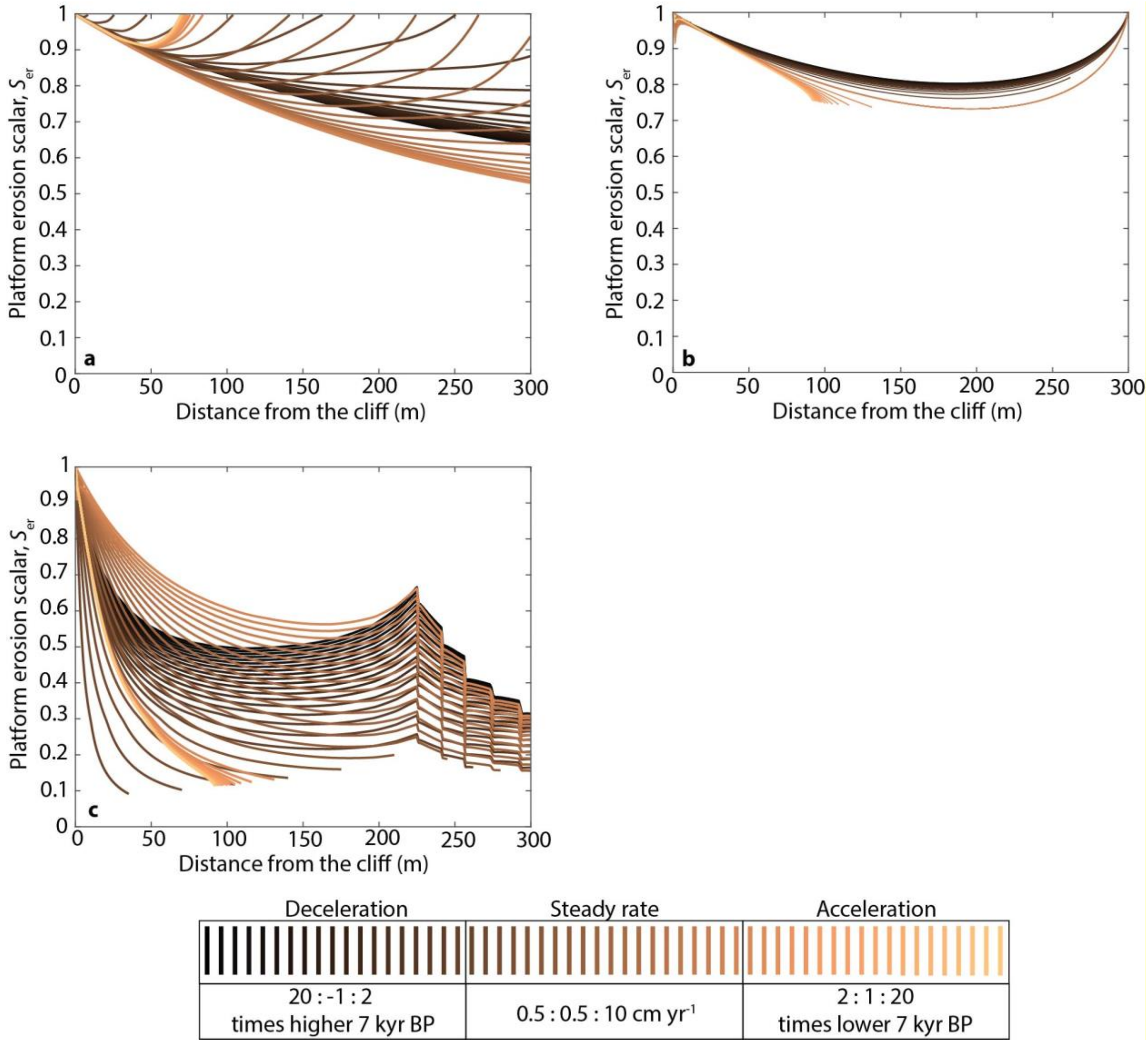

Supplementary Figure 3. Results of modelling total platform erosion scalar across the 300 m profile (Fig. 1 in the main document) under 58 scenarios of cliff retreat and: a) profile-parallel erosion model; b) platform widening; c) empirically-based model of platform down-wearing. Specific cliff retreat scenarios are consistently coloured and explained in the bottom colour-scheme plot.

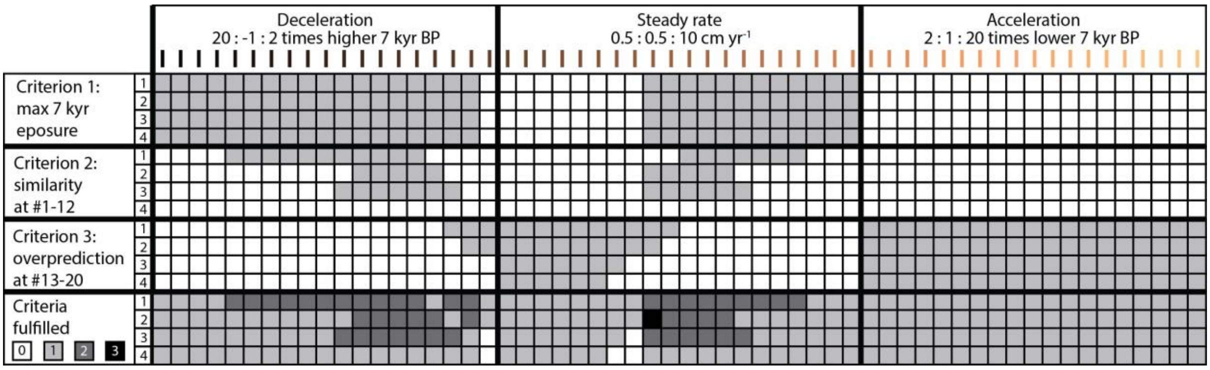

Supplementary Figure 4. Fulfilment of the three criteria imposed onto 232 scenarios that combine different rates and modes of cliff retreat and shore platform down-wearing; platform erosion models: 1 – zero platform down-wearing, 2 – profile-parallel erosion model, 3 – platform widening, 4 – empirically-based model of platform down-wearing. The colour code above the grid refers to the cliff retreat rates scenarios of other figures in the main and supplementary documents.

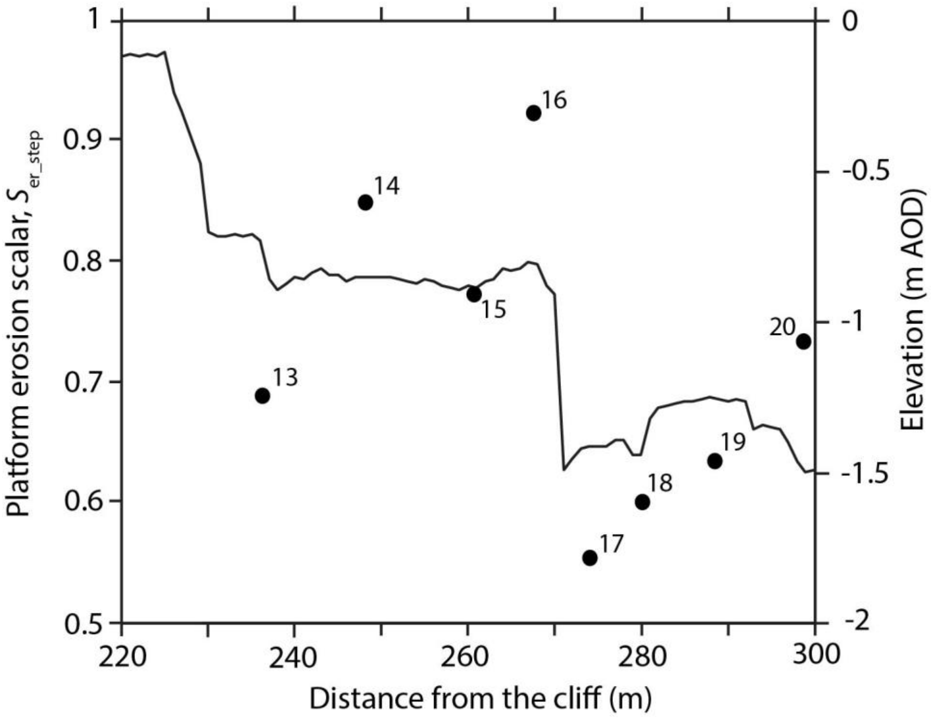

Supplementary Figure 5. Platform erosion scalar due to the step back-wearing contribution, S_{er_step} (points) at the seaward section of the Hartle Loup platform; the line represents the distribution of elevation.

SUPPLEMENTARY REFERENCES

1. Corbett, L. B., Bierman, P. R. & Rood, D. H. An approach for optimizing in situ cosmogenic 10Be sample preparation. *Quat. Geochronol.* **33**, 24-34 (2016).
2. Hurst, M. D., Rood, D. H., Ellis, M. A., Anderson, R. S. & Dornbusch, U. Recent acceleration in coastal cliff retreat rates on the south coast of Great Britain. *Proc. Natl. Acad. Sci. U.S.A.* **113(47)**, 13336-13341 (2016).
