# Supplementary material for: Cosmogenic exposure dating reveals limited long-term variability in erosion of a rocky coastline
Source: Nat Commun. 2020 Jul 30;11:3804. doi: 10.1038/s41467-020-17611-9 (PMC7393086; doi:10.1038/s41467-020-17611-9)
Supplement: Supplementary file 2 — Description of Additional Supplementary Files [file 41467_2020_17611_MOESM2_ESM.pdf]

Description of Additional Supplementary Files

File Name: Supplementary Data 1
Description: The calculation of ^{10}Be concentrations in the samples based on the sample weight and the AMS $^{10}Be/^{9}Be$ ratios.

File Name: Supplementary Data 2
Description: Numerical model and input matrices used to explore cosmogenic ^{10}Be concentrations and reconstruct the long-term rocky coastline erosion.
